# Progress toward Sustainable Development Goals and interlinkages between them in Arctic countries

**DOI:** 10.1016/j.heliyon.2023.e13306

**Published:** 2023-01-31

**Authors:** Qiang Bie, Shijin Wang, Wenli Qiang, Xing Ma, Zhengsheng Gu, Nan Tian

**Affiliations:** a Faculty of Geomatics, Lanzhou Jiaotong University, Lanzhou 730070, China; b Yulong Snow Mountain Cryosphere and Sustainable Development Field Science Observation and Research Station/State Key Laboratory of Cryospheric Sciences, Northwest Institute of Eco-Environment and Resources, Chinese Academy of Sciences, Lanzhou 730000, China; c College of Earth and Environmental Sciences, Lanzhou University, Lanzhou, 730000, China; d School of International Relations, Liaoning University, Shenyang, 110136, China

**Keywords:** Composite index, SDGs, Measuring progress, Interlinkages, Synergies and trade-offs, Arctic countries

## Abstract

The adoption of the 2030 Agenda and its 17 Sustainable Development Goals (SDGs) contribute to addressing the multiple and complex challenges faced by humankind. In particular, the increasing impact of climate change and globalization represent a great challenge for the sustainable development of Arctic countries and efforts are needed to measure, assess, and compare the progress toward SDGs and the trends in this region. This study used 69 indicators closely related to Arctic countries and a composite indicator approach to assess their sustainability performance. SDG interlinkages were also assessed through Spearman's rank order correlation. The results showed that the sustainable development of Arctic countries gradually improved from 2000 to 2020, with increasing scores recorded for 82% of the goals and 73% of the indicators. Overall, significant progress was observed in the SDG 10 (reduction of inequality), SDG 3 (health improvement), and SDG 4 (quality of education). The highest-ranking scores were obtained for poverty reduction (SDG 1), SDG 3, and climate actions (SDG 13) in 2020. Over the 21-year period, Sweden reached the highest overall score for sustainable development, and Russia showed the greatest improvement. Synergies dominated over trade-offs among and within SDGs. SDGs 1, 3, 9, 10, and 11 presented a relatively higher proportion of synergies, while higher proportions of trade-offs were observed for the 8–9, 8–11, 3–12, and 10–12 SDG pairs. The associations of industry development with clean energy use and environmental conservation were strengthened during the study period. However, the performance varied greatly for different indicators, goals, and their correlations among Arctic countries. The results indicate that the main challenges for these countries in relation to SDGs consist in promoting an inclusive economic model as well as sustainable consumption and production patterns.

## Highlights

• A synthetic indicator and composite method was applied in assessing progress and interlinkages in regional SDGs' achievement.
• Obvious progress was observed in inequality reduction, health improvement and climate actions in Arctic countries.
• Synergies dominated over trade-offs among and within SDGs in Arctic countries.
• The performance varied greatly for different indicators, goals, and their correlations among Arctic countries.

## Graphical abstract

**Figure:**
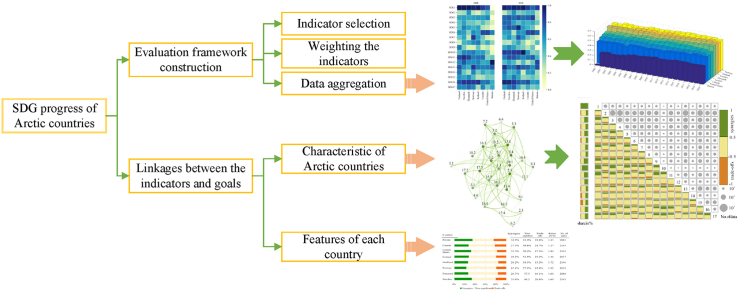

## 1 Introduction

Sustainable development has become an important topic in connection with the numerous environmental challenges currently faced by humanity [1]. The term “sustainable development” was mentioned in the world Charter of nature in 1982, which was elaborated and reached a global consensus at the United Nations Conference on environment and development in Rio in 1992 [2]. The United Nations (UN) proposed the millennium development goals to address the challenges of extreme poverty, hunger, illiteracy, and disease, and then adopted the 2030 Agenda for Sustainable Development Goals (SDGs) in 2015, which included 17 goals and 169 targets. The SDGs provide a comprehensive and multidimensional framework to assess and solve the social, economic, and environmental issues related to development in an integrated manner between 2015 and 2030 and, specifically, to move toward a sustainable development path [3]. Reaching the SDGs will require deep transformations in both developing and developed countries [4]. It is also necessary to assess the progress made in achieving the goals at both the regional and global scales in order to identify and address critical development issues or potential gaps [1,5].

Focusing on the framework of the 2030 Agenda, governments and researchers are currently measuring and monitoring the progress toward SDGs at different geographical scales, using different indicators and composite methods. The UN used 122 indicators for 193 countries to analyze the achievement of SDGs globally between 2000 and 2021. Kroll (2015) [3] examined and compared the performance toward SDGs in high-income countries throughout the 2000–2015 period using 34 indicators and various studies assessed the achievement of SDGs in the European Union (EU). For example, Lafortune et al. (2021) [6] selected 107 indicators and used the UN framework to detect the progress of the region toward sustainable development; Bolcárová and Kološta (2015) [7] used 12 indicators and the aggregated index to measure the sustainable development of each of the 27 EU countries between 2004 and 2007; and Carrillo (2022) [8] proposed a novel composite indicator approach based on 64 indicators to assess the EU’s performance during 2010 and 2020. For 15 countries along the Belt and Road region, the achievement of SDGs was assessed using 108 indicators [9]. Other studies evaluated SDGs in the Arab region [10], central Asia [11], Asia-Pacific countries [12], and southeastern Europe [13]. In addition, specific SDGs were monitored, such as those related to health [14,15], economy (SDG8), consumption (SDG 12) [16], and climate impacts (SDG13) [17].

Previous studies have proved that indicators play an instrumental role in the definition of sustainable development, and found that the countries’ relative position in terms of performance toward SDGs almost entirely depends on the chosen method of assessment and indicators [[18], [19], [20]]. Thus, without a procedurally well-designed conceptual framework to select or design indicators, the results of SDG assessments may be ambiguous and confusing [21,22]. The SDG approach is based on a set of objectives which should help improve the situation of poor and developing countries [13]. As different countries and regions present vary greatly in terms of resources and environmental background, social and economic development, and development stage overall, they may have different association mechanisms to meet the SDGs [23].

As the targets of SDGs are multi-dimensional and integrated with each other, the progress toward one target is also linked through complex feedbacks to other targets [24,25]. This interrelationship can be divided into two types: synergy and trade-off, where progress toward one goal favors or hampers the progress toward another goal, respectively. Quantitative analysis of the interactions between different SDGs (which is one of the most urgent SDG-related research priorities) can reveal their nature and provide policy makers with more detailed information [24]. Previous studies have also proved that SDG interactions vary based on region and country's income along with the gender, age, and location of its population [26].

Therefore, it is necessary to establish sustainable development frameworks at the regional scale [19,27]. Arctic region gained the attention globally for its economic, political and ecological importance at the background of global climate change [28]. In particular, due to the unique geographical location and fragile ecology of the Arctic region, which has also experienced rapid environmental change and has become the object of increasing industrial interests [29], there has been a call for achieving SDGs in Arctic countries than the global perspectives [30]. In addition, the difference between Arctic countries is significant in relation to the economic, natural resources and other aspects.

Although the existing studies have focused on the application of global SDGs in the Arctic and added the specific indicators for the Arctic, such as indicators about indigenous rights, livelihoods and knowledge systems [31,32]. However, these studies were all stayed in the concept stage [30,31], for the quality measurement was still lacked for the data availability at the regional scale. Thus, for the data availability, we selected the Arctic countries as our study area.

Thus, the aim of this study was to construct a synthetic indicator and composite method to evaluate the performance of Arctic countries toward sustainable development, establish how far is each country from achieving specific SDGs, and how do the countries differ in their adoption of the goals. In addition, the study analyzed how trade-offs and synergies between indicators and goals.

## 2 Methodology and data

### 2.1 Constructing the indicator framework and database

#### 2.1.1 Indicator selection

For each goal and its corresponding targets, the most representative and important indicators associated with Arctic countries (including the United States, Canada, Russia, Denmark, Sweden, Iceland, Finland and Norway) were selected by searching relevant publications that included the major targets and indicators of the SDGs established by the UN as well as the indicators reported in other studies, such as those used to evaluate the sustainability of the EU, high income countries, and Organization for Economic Co-operation and Development (OECD) countries.

To assess the sustainability of *n* countries, a number *d*_*i*_^*k*^ of simple indicators was selected within each *k* dimension. The whole dataset to be subjected to analysis was then gathered in a *n* x *m* matrix, whose $d_{ij}^{k}$ elements measured the performance of the *i*-th indicator of year *j* in relation to the in the *k-th* sustainability goal (*i = 1, …, d*_*k*_*, j = 1, …, n, k = 1, …, 17*).

The indicators were categorized as positive and negative based on their attributes. For the indicators that the higher value means the more sustainable, which stands for higher observed values imply a superior performance in the corresponding sustainability dimension, and set *P* for these positive indicators. While set N for the negative indicators that the lower values present better performance.

#### 2.1.2 Data normalization

The observed data needed to be normalized due to the differences in measurement and unit dimension for different indicators. The linear extreme value method was used to normalize the raw data in this study, with their values varying between 0 and 1. Then, Eq. (1) was used for the positive indicator (the higher the indicator value, the better the sustainability performance) and Eq. (2) for the negative indicator (the lower the indicator value, the worse the sustainability performance). The equations are defined as:

(1) $$D_{ij}=\frac{d_{ij}^{k}-d_{i}^{\min}}{d_{i}^{\max}-d_{i}^{\min}}$$

(2) $$D_{ij}=\frac{d_{i}^{\max}-d_{ij}^{k}}{d_{i}^{\max}-d_{i}^{\min}}$$

where $D_{ij}$ is the normalization value of indicator *i* in the year *j*, $d_{ij}^{k}$ represents the observed value of indicator *i* in the year j of goals k, and $d_{i}^{\min}$ and $d_{i}^{\max}$ are the minimum and maximum values for indicator *i* over the study period.

#### 2.1.3 Weighting the indicators

The weight of an indicator is a quantitative expression of its importance relatively to other indicators used in the assessment. The equal-weight scheme is rather common in practical applications, but it masks a different weighting pattern when the number of indicators is uneven across different dimensions. The expert-based weights are also widely used to account for different theoretical backgrounds, but they can be influenced by the experts’ academic background or geographic characteristics.

In this study, an objective computation of indicator weights that uses the standard multi-criteria decision making (MCDM) closeness-to-an-ideal paradigm was selected, as described in Ref. [8]. The computation of weights is based on the fact that countries are more similar in their performance to a utopian (ideal) country that would be considered the most sustainable, defined as one showing the best indicator performance within a given dimension. Thus, all the other countries would minimize the distance between their own performance and the ideal performance.

The minimization of the *n* distances for each indicator is then formalized as a multi-objective optimization problem, and these minimum distances are approached by minimizing the sum of distances of all the evaluated units to that of the ideal unit. Then, using the weighted Euclidean distance, a set of neutral weight values is obtained through the following quadratic programming problem, which must be solved to obtain the *w*_*j*_^*k*^ indicator weights for the *m*_*k*_ indicators within each SDG (k = 1, …17), shown in Eq. (3):

(3) $$\begin{array}{c} \min_{s.a}\;\sum_{i=1}^{d_{k}}{\left(w_{i}^{k}\left(D_{ij}^{k}-D_{ij}^{k,U}\right)\right)}^{2}\\ \sum_{i=1}^{d_{k}}w_{i}^{k}=1\\ w_{i}^{k}\geq 0 \end{array}$$

where $D_{ij}^{k,U}$ is the maximum value of indicator *k* in goals j, and $D_{ij}^{k}$ is the value of indicator *k* in goals *j*. The indicator weighting results are shown in Table 1.

**Table 1:** List of indicators, their attribute (Negative(N)/Positive(P)), and aggregation weights.

SDG1. No Poverty	N/P	W	SDG9. Industry, Innovation, and Infrastructure	N/P	W
Poverty headcount ratio at $3.20 a day (N-0-100)	N	0.50	Research and development expenditure (% of GDP)	P	0.20
Poverty rate after taxes and transfers	N	0.40	Researchers in R&D (per million people)	P	0.50
Poverty headcount ratio at $5.50 a day	N	0.10	The Times Higher Education Universities Ranking: Average score of top 3 universities (worst 0–100 best)	P	0.10
**SDG2. Zero Hunger**			Scientific and technical journal articles (per 1000 people)	P	0.20
Prevalence of obesity among adults	N	0.05	**SDG10. Reduce Inequalities**		
Human Trophic Level (best 2–3 worst)	N	0.20	Gini coefficient	N	0.50
Cereals, Total-yield (t/ha)	P	0.15	Inequality-adjusted HDI	P	0.50
Agriculture, forestry, and fishing, value added per worker	P	0.25	**SDG11. Sustainable Cities and Communities**		0.20
Agriculture area under organic	P	0.10	Annual mean concentration of PM2.5 (μg/m³)	N	0.50
Arable land (% of land area)	P	0.25	Satisfaction with public transport (%)	P	0.20
**SDG3. Good Health and Well-Being**			Improved water source, piped (% urban population with access)	P	
Life satisfaction	P	0.10	Urban population (% of total population)	P	0.10
Current health expenditure (% of GDP)	P	0.40	**SDG12. Responsible Consumption and Production**		
Life expectancy at birth, total (years)	P	0.10	E-waste generated (kg/capita)	N	0.40
Rate due to cardiovascular disease, cancer, diabetes, or chronic respiratory disease in adults	N	0.30	Material consumption (tonnes per capita)	N	0.40
Traffic deaths (per 100,000 population)	N	0.10	Municipal solid waste (kg/capita/day)	N	0.20
**SDG4. Quality Education**			**SDG13. Climate Action**		
School enrollment, tertiary (% gross)	P	0.10	CO2 emissions (kg per 2015 US$ of GDP)	N	0.10
Adult participation in learning	P	0.30	CO2 emissions (metric tons per capita)	N	0.20
Mean years of schooling	P	0.20	Climate Change Vulnerability Monitor (best 0–1 worst)	N	0.10
Current education expenditure, total	P	0.20	Total Damages affected by disasters (% GDP)	N	0.50
PISA score (worst 0–600 best)	P	0.20	Population covered by the Covenant of Mayors for Climate & Energy signatories	P	0.10
**SDG5. Gender Equality**			**SDG14. Life Below Water**	P	
Ratio of female-to-male mean years of education received	P	0.20	Ocean Health Index	N	0.30
Ratio of female to male labor force participation rate	P	0.50	Percentage of fish stocks overexploited and collapsed by exclusive economic zone	P	0.40
Gender wage gap	N	0.20	Mean area that is protected in marine sites important to biodiversity (%)	P	0.30
Proportion of seats held by women in national parliaments	P	0.10	**SDG15. Life on Land**		
**SDG6. Clean Water and Sanitation**			Annual change in forest area (%)	N	0.20
People using safely managed drinking water services	P	0.10	Red List Index	N	0.10
Population using at least basic sanitation services	P	0.20	Share of forest area	P	0.20
Virtual water dependency on rest of the world	N	0.50	Mean area that is protected in terrestrial sites important to biodiversity (%)	P	0.40
Freshwater withdrawal as a proportion of available freshwater resources	N	0.20	Mean area that is protected in freshwater sites important to biodiversity (%)	P	0.10
**SDG7. Affordable and Clean Energy**			**SDG16. Peace, Justice, and Strong Institutions**		
Energy imports, net	N	0.30	Homicides (per 100,000 population)	N	0.10
CO_2_ emissions from fuel combustion per total electricity output	N	0.50	TIV of arms exports to all	N	0.50
Share of renewable energy in total primary energy supply	P	0.10	Corruption Perceptions Index (worst 0–100 best)	P	0.20
Energy use (kg of oil equivalent per capita)	N	0.10	Government Efficiency (1–7)	P	0.20
**SDG8. Decent Work and Economic Growth**			**SDG17. Partnerships for the Goals**		
GDP growth (annual %)	P	0.20	Foreign direct investment, net inflows (% of GDP)	P	0.20
Unemployment, total (% of total labor force)	N	0.50	Export of goods and services (% of GDP)	P	0.40
Net investment in nonfinancial assets	P	0.20	Official development assistance as a percentage of GNI	P	0.10
Total natural resources rents	P	0.10	trade links	P	0.30

#### 2.1.4 Data aggregation

The SDG subindex and the composite sustainability index were constructed to evaluate the comprehensive performance toward SDGs in Arctic countries over time, and the process included two steps. Firstly, the SDG subindex *GCI*_*k*_ (k = 1,…, 17) was constructed to reflect the overall performance of each country toward different goals and, by aggregating the *D*_*k*_ indicators within the *k-th* sustainability dimension, the 17 goal composite indicators were obtained. The subindex was constructed using Eq. (4) as follows:

(4) $${GCI}_{ij}^{k}=\sum_{i=1}^{d_{k}}w_{i}^{k}D_{ij}^{k}$$

Secondly, the 17 indexes obtained for each sustainability dimension considered were aggregated into the sustainability index (SI) and the geometric average was then selected. The SI was constructed using equation (5), as follows:

(5) $${SI}_{ij}^{k}=\prod_{j=1}^{17}{\left({GCI}_{ij}^{k}\right)}^{\frac{1}{17}}$$

#### 2.1.5 Assessing linkages between SDGs

Numerous interactions exist among indicators, which means that, in order to progress toward SDGs, it is necessary not only to improve the performance of each indicator, but also to pay attention to the relationships between them. The present study aimed to identify the interlinkages between indicators while measuring progress toward the SDGs, and also to shed light on the extent to which synergies and trade-offs facilitate or hinder countries in moving toward the 2030 Agenda's objectives. In addition, the interlinkages between different goals were established at both the regional and country levels to explain differences between countries in the achievement of SDGs.

In this study, the synergies and trade-offs were evaluated statistically based on the significance of positive and negative correlations. Spearman's rank correlation has been used to assess interlinkages between SDGs in previous studies [33,34] and it was also employed here as it can reveal the non-linear relationships between variables and is less sensitive to outliers. Our analysis only considered data pairs containing more than three data points to avoid potential false detection. As in other studies, a threshold of ±0.5 was used for the correlation coefficient to define a synergy or a trade-off between two indicators in a pair. The relation was considered as strong or moderate if it was above or below this threshold, respectively, and the correlation was considered significant if its p-value was below 0.1 [3,26]. Thus, a correlation indicated a synergy if the correlation coefficient (Spearman's rho) was above 0.5 and a trade-off if it was below −0.5 [3]. Indicator pairs with a correlation coefficient between −0.5 and 0.5 were labelled as non-correlations. The correlation results for each country were then aggregated by calculating the overall percentage of synergies, trade-offs, and non-correlations for Arctic countries as a whole.

## 3 Results

### 3.1 Characteristic of indicators

Economic development is a key dimension of the SDGs. Most indicators related to the economy of Arctic countries showed promising results, such as those in SDG1. In particular, the data of three indicators for this goal, i.e., Target 1.1, 1.2, and 1.3, exhibited a decreasing trend from 2000 to 2020 in all the eight Arctic countries examined, especially in Russia, where their values decreased by 98.0%, 59.0%, and 92.0%, respectively. Although there were still large disparities among countries, the gap was reduced during the study period. The highest value for GDP growth was observed in Russia at 9.8% in 2000, and then observed in Iceland at 2.4% in 2020. Finland and Iceland had the highest and lowest unemployment rates in 2000, recorded at 11.1% and 1.9%, respectively, while in 2020 the highest value was observed in Canada at 9.5%. The trends of GDP growth and employment tended to increase during the 2000–2019 period, but they reversed in 2020 due to the COVID-19 pandemic.

Natural resource endowment was also important in the analysis of sustainable development. In 2000, Russia and Iceland had the largest and smallest natural resources rents, recorded at 22.0% and 0%, respectively. Finland showed the highest values for energy import dependency (53.9% in 2000 and 41.0% in 2020), whereas the values recorded in Iceland and Sweden decreased from 35.8% to 6.56%, and from 22.6% to 14.5%, respectively, during the 2000–2020 period. Denmark switched from being a net energy exporter to a net importer during the study period. The share of forest area in the eight Arctic countries was above 30.0% in 2020, except in Iceland and Denmark, and Finland showed the highest value of 73.8% for this indicator in 2000.

The data obtained from the indicators related to agriculture, such as cereal yield, agriculture output per worker, and proportion of organic agriculture area, also increased during the study period (except in 2020). However, the indicators related to the “zero hunger” goal presented the opposite trend, with the prevalence of obesity among adults in all eight countries increasing by nearly 50.0% from 2000 to 2020. In 2020, the highest values were observed in the United States (38.6%) and Canada (31.8%), and the lowest in Russia (22.1%) and Denmark (21.3%), and the disparity between countries also increased.

At the same time, all the values for the health and well-being indicators showed a promising trend, especially for health expenditure, whose average value in the eight countries increased from 8.1% to 10.4%, with the highest and lowest observations recorded in the United States and Russia, respectively. Sweden showed the largest growth rate (47.0%). The rate of mortality due to cardiovascular disease, cancer, diabetes, or chronic respiratory disease in adults decreased from 17.9% to 11.4%. The highest values were recorded in Russia (37.0% in 2000 and 23.1% in 2020) and the lowest in Sweden (13.2% in 2000 and 8.2% in 2020). The gap between the highest and lowest observations was closed.

For the indicators related to education and innovation, the average proportion of tertiary school enrollment increased from 64.1% to 85.8% in the eight Arctic countries. Finland reached the highest value of 95.6% in 2020, while Sweden recorded the best achievement of adult participants in learning at 29.3% in 2019. Huge disparities were also observed in investments on Research & Development (R&D); the highest and lowest expenditures were observed in Sweden and Russia with about 3.1–4.1% and 1.0–1.3% of the GDP, respectively, the latter corresponding to only half of the average of Arctic countries. In 2020, Finland showed the largest number of researchers in R&D (9003 per million people), followed by Iceland (6527 per million people).

The indicators of environmental dimensions also showed large variations, especially those related to climate actions. The highest value of CO_2_ emissions per capita was observed in the United States in 2000 (20.5 Mt), while the lowest was recorded in Sweden in 2020 (3.3 Mt). In nearly all the Arctic countries examined, the values of this indicator during the 2000–2020 period decreased by approximately 9–54%, except in Russia, where they increased by 13%. The CO_2_ emissions from fuel combustion per total electricity output also declined by 16–68% during the study period in almost all the Arctic countries, except in Norway where it increased by 31%. In 2020, the minimum value was observed in Iceland (0.09 Mt) and the maximum values in Russia (1.6 Mt) and the United States (1.2 Mt). Iceland also produced the largest amount of renewable energy, which increased from 77.4% to 88.9% during the study period, while, in 2020, Russia and the United States accounted for the lowest production, which was only 2.6% and 8.0%, respectively. Material consumption and waste generated per capita were the lowest in Russia and the highest in Canada and the United States. In addition, Denmark recorded the largest volume of electrical waste per capita.

### 3.2 Comprehensive performance toward the achievement of SDGs

Based on the assessment results, the Arctic countries examined have progressed toward 14 SDGs over the past 20 years. The average scores indicated that significant progress has been achieved for SDG 3 (*Good health and well-being*), SDG 4 (*Quality of education*), SDG 5 (*Gender equality*), SDG 9 (*Industry, innovation, and infrastructure*), and SDG 10 (*Reduction of inequality*), whose values increased by 35.9%, 42.4%, 43.9%, 37.3%, and 39.5%, respectively, during the 2000–2020 period. In contrast, the scores of SDG 8 (*Decent work and economic growth*), SDG 12 (*Responsible production and consumption*), and SDG 17 (*Partnerships that promote the achievement of goals*) showed a downward trend, decreasing from 0.48, 0.68, and 0.57 to 0.35, 0.47, and 0.53, respectively.

The relative scores of different goals also varied significantly over time. In 2000, the mean values of SDG 1 (*No poverty*), SDG 11 (S*ustainable cities and communities*), and SDG 16 *(Peace, justice, and strong institutions*) were relatively higher, while in 2020, the scores of SDG 1 (*No poverty*), SDG 3 (*Good health and well-being*), and SDG 13 (*Climate action*) were ranked as the top three overall, indicating that Arctic countries are making efforts to improve nutrition, health, and well-being, and are taking actions to cope with climate change. The worst performances were those toward SDG 8 (*Decent work and economic growth*), SDG 2 (*Zero hunger*), and SDG 12 (*Responsible production and consumption*).

**Fig. 1:**
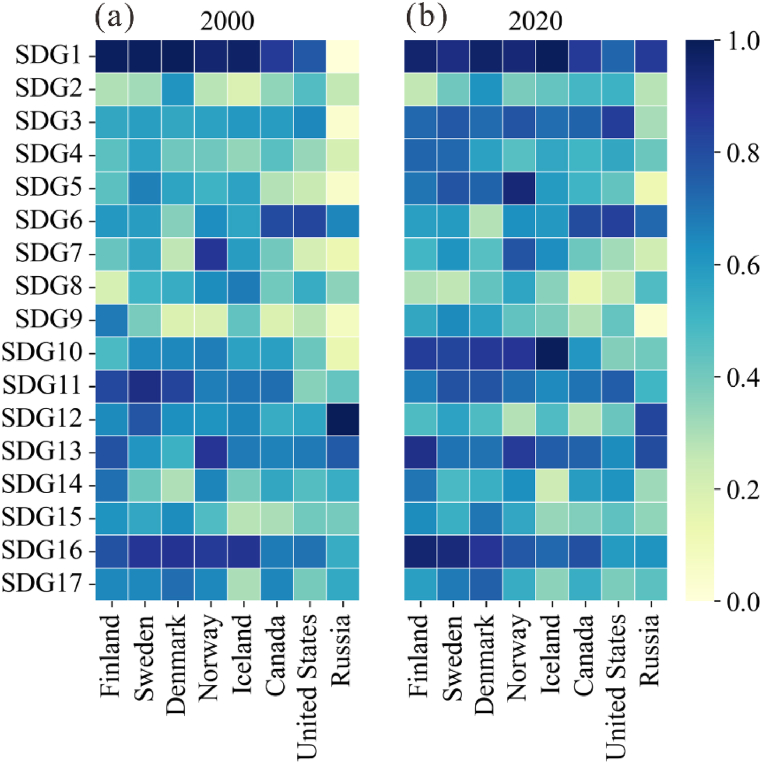
Scores of the 17 SDG goals for each Arctic country in 2000 (a) and 2020 (b).

Significant differences were detected in the performance of countries toward different goals over the 2000–2020 period (Fig. 1). In terms of the economy-related goals (i.e., SDG 1, SDG 8, and SDG 9), Denmark, Finland, and Sweden achieved the highest scores for SDG 1 in 2000 (0.99) and Russia showed great progress (from 0 to 0.86), while, in 2020, the scores achieved by Sweden, the United States, and Finland showed a slight downward trend, and those obtained by the United States were the lowest (Fig. 1a). During the 2000–2020 period, Norway overtook Iceland as the best performer toward SDG 8. The values of this goal in all the Arctic countries showed a sharply decreasing trend between 2019 and 2020, with Iceland experiencing the largest decline, while Russia and Finland recorded more limited reductions (Fig. 1b). However, the lowest values of SDG 8 in most countries were observed in 2009 and they are attributed to the economic crisis. During the study period, Sweden overtook Finland as the best performer toward SDG 9. Denmark, Norway, Sweden, and the United States showed remarkable progress toward this goal as well, while the performance of Finland, Iceland, and Russia slightly decreased.

The scores of SDG 2 (*Zero hunger*) and SDG 3 (*Health and well-being*) showed a promising trend. For the former goal, Denmark had the highest score and maintained its leading position, while Iceland and Canada made significant progress with scores increasing by 0.24 and 0.15, respectively. The best performance toward SDG 3 was reported in the United States, followed by Sweden and Norway. Russia showed the worst performance toward this goal but recorded the largest increase with 0.27 of all eight countries. The difference between the maximum and minimum values of SDG 3 in 2000 was 0.60 and decreased to 0.53 in 2020.

Progress was also achieved in the efforts to reduce inequality and improve the quality of education. Both SDG 5 (*Gender equality*) and SDG 10 (*Reduction of inequalities*) displayed a positive trend in all eight countries during the 2000–2020 period. Sweden showed the best performance toward SDG 5 in 2000, and its leading position was taken by Norway in 2020, while Russia, the United States, and Canada were ranked at lower positions. For SDG 5, the disparity between different countries expanded from 0.61 to 0.82. Iceland showed the greatest progress toward SDG 10 in 2020, and Norway, Sweden, Denmark, and Finland also relatively improved their position in the achievement of this goal. In contrast, during the study period, a downward trend was noted for the United States, which were ranked at the last position. Sweden showed the best performance toward SDG 4 (*Quality of education*) in 2000, and Finland experienced the largest increment of 0.28, reaching the highest-ranking position in 2020. The United States, Iceland, and Russia also remarkably improved their performance toward SDG 4.

Arctic countries also made significant efforts to facilitate the transition toward a carbon-neutral economy and address the challenge of climate change. Nearly all countries improved the aggregate performance toward SDG 7 (*Affordable and clean energy*) and SDG 13 (*Climate actions*). During the study period, Norway was ranked first and had a relatively higher position in terms of progress toward SDG 7 and SDG 13, respectively, mainly due to its relatively lower energy use and carbon emissions per capita and higher proportion of renewable energy. Iceland was ranked second as it has a lower proportion of energy imports and higher share of renewable energy. Denmark showed the greatest improvement in its performance toward SDG 7 and SDG13, while Russia and the United States showed the worst performances toward these two goals, respectively.

Russia retained the leading position in terms of progress toward SDG 12 (*Responsible consumption and production*), while Canada was the worst performer. The scores obtained by all countries presented a downward trend mainly due to the increase of material footprint and waste generated. Finland and Denmark showed the best performance toward SDG 14 (*Life below water*) and SDG 15 (*Life on land)*. Denmark and the United States progressed remarkably toward SDG 14, while Iceland and Russia showed downward trends. All countries showed a slight change of SDG 15. Finland recorded the greatest improvement toward SDG 16 (*Peace, justice, and strong institutions*) during the study period, moving from the third to the first position in 2020. Denmark and Iceland were ranked first and last, respectively, in terms of progress toward SDG 17 (*Partnerships for the goals*), and both Canada and Norway showed a downward trend.

**Fig. 2:**
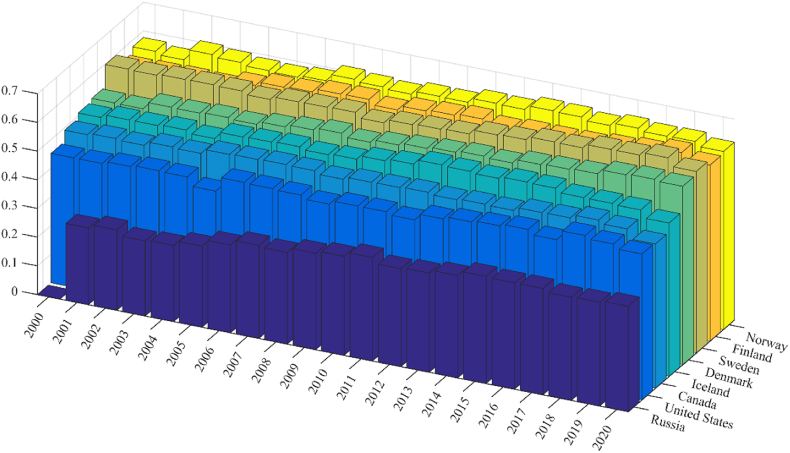
Composite Sustainability Index (GSI) values of SDGs for Arctic countries during the 2000–2020 period.

Sweden showed the highest GCI value over the study period, and Norway (ranking second in 2000 and third in 2020) and Finland (ranking third in 2000 and fourth in 2020) also maintained relatively high positions (Fig. 2). The United States moved up from the seventh position in 2000 to the sixth in 2020. The positions of Sweden, Iceland, and Russia did not change. During the study period, the highest improvement rate in the GCI was experienced by Russia, even if this country was still ranked in the last position. The second-best improvement rate during the 2000–2020 period was observed in Denmark, which moved up from the fourth position in 2000 to the second position in 2020. The GCI values presented an upward trend in all eight Arctic countries, with an average growth rate of 18.8%. The dispersion between the highest and lowest values was sharply reduced from 0.60 to 0.26, which means that the difference decreased between countries in sustainability development.

### 3.3 Synergies and trade-offs between SDGs

**Fig. 3:**
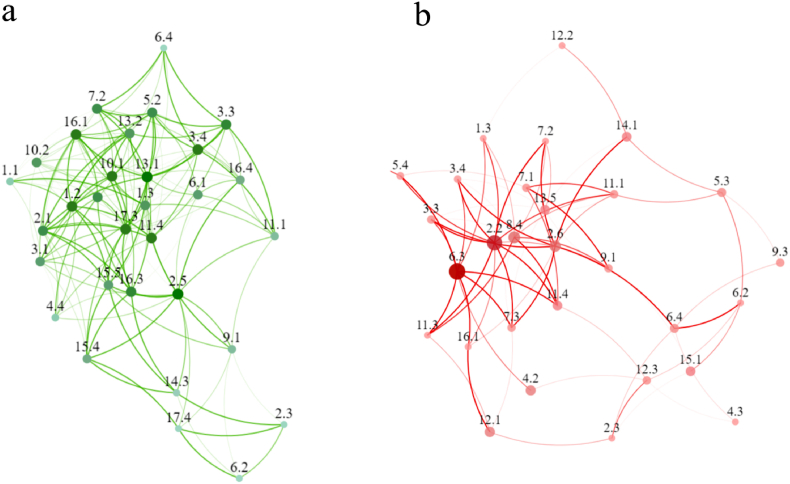
Network analysis of positive interlinkages (a) and negative interlinkages (b) between SDG indicators for the Arctic countries examined. （Darker green color represents higher outdegree score. Larger icon represents higher closeness centrality score）. (For interpretation of the references to color in this figure legend, the reader is referred to the Web version of this article.)

Fig. 3 shows the results of the network analysis applying the positive (Fig. 3a) and negative (Fig. 3b) interlinkages of indicators to the Arctic countries. The SDG indicator of 1.2 (*Poverty rate after taxes and transfers*) showed the largest proportions of positive correlations, reporting 25 pairs of synergies with other indicators, and the correlations for 11 of these pairs were positive and above 0.5 over the 21-year period. Indicator 2.1 *(Prevalence of obesity among adults, BMI≥30)* showed the second highest positive correlations with other indicators, and the indicator of 2.5 (*Agriculture area under organic*) and 3.5 (*Traffic deaths*) also showed a relatively larger number of positive relations of 21, followed by 1.3 (*Poverty headcount ratio at $5.50 a day*), 3.3 (*Life expectancy at birth*), and 3.4 (*Age-standardized death rate due to cardiovascular disease, cancer, diabetes, or chronic respiratory disease in adults*), with the positive relationships of 21. In addition, the indicator of 5.4 (*Proportion of seats held by women in national parliaments*) and 5.2 (*Ratio of female to male labor force participation rate*) indicators showed even more positive links with other indicators. In particular, this was the case for SDG 1, 3 and 5. Similarly, 13.1 and 13.2, which are related to carbon emissions, also showed larger positive links with other indicators.

While for the negative interlinkages of the indicators (Fig. 3b), The SDG indicators of 6.3 (Virtual water dependency on rest of the world) and 2.2 (Human trophic level) ranked the largest second negative proportion of the negative correlations. Indicator 6.3 showed large proportions of negative correlations with the indicators for SDG 1, 3, 9 and 16. The indicator of 2.2 displayed the larger proportion of negative correlations with indicator of 1.2 (*Poverty rate after taxes and transfers*), 3.3 (*Life expectancy at birth*), 3.4 *(Rate due to cardiovascular disease, cancer, diabetes, or chronic respiratory disease in adults),* 11.4 *(Urban population) and* 13.1 *(CO2 emissions),* with the largest negative relationships of 21.

**Fig. 4:**
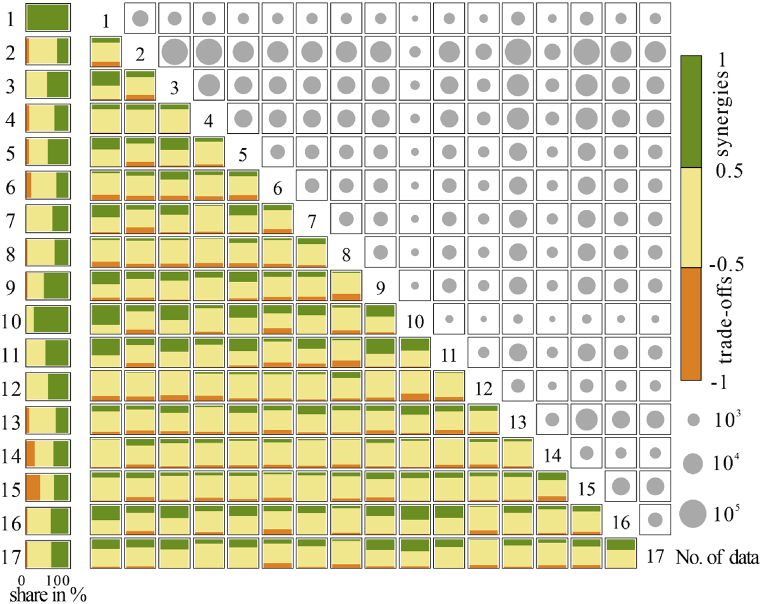
Interactions between sub-indicators within (left) and between (right) SDGs (the color bars represent the shares of trade-offs (orange), synergies (green), and non-classified interactions (yellow) in Arctic countries. The numbers in boxes represent the number of data pairs used for each analysis). (For interpretation of the references to color in this figure legend, the reader is referred to the Web version of this article.)

The overall results of the interactions within each SDG indicated that, during the 2000–2020 period, synergies largely outweighed trade-offs (Fig. 4). The variations between goals were obvious. SDG1 (*No poverty*), 3 (*Good health and well-being*), 9 (*Industry, innovation, and infrastructure*), 10 (*Reducing inequality*), and 11 (*Sustainable cities and communities*) showed a larger proportion of synergies compared to other goals, with *p* values above 0.5 for 60–90% of data pairs, which means that the improvement of one indicator was accompanied by the improvement of another indicator in these goals. In contrast, SDG 6 (*Clean water and sanitation*), 14 (*Life below wate*r), and 15 *(Life on land*) showed a relatively higher proportion of trade-offs with 13–34% of data pairs of all. These can be identified by the significant correlation between the indicators of these goals; for instance, the improvement of indicator 6.2 (*Population using at least basic sanitation services (%)*) was associated with the expansion of freshwater withdrawal as the proportion of available freshwater resources (%), with a significant negative correlation of −0.62. The synergies between the indicators of most goals were relatively stable over time. The exceptions were SDG 5 and SDG 9, which presented a lower proportion of synergies from 2000 to 2006; however, for the former, this proportion increased since 2016, indicating the successful implementation of the Agenda 2030. In contrast, the proportion of synergies in SDG 9 showed a continuous slightly downward trend, suggesting that further improvements are needed in terms of innovations for sustainable development going forward.

Both positive and negative correlations were observed among different goals (Fig. 4). SDG 1 showed the largest number of synergetic relationships with other goals on average during the study period and it was statistically linked with progress in SDG 3, SDG 5, SDG 7, SDG 9, SDG 10, and SDG 11 with 40–70% of the data pairs. Numerous synergies were observed also for SDG 3, whose improvement was associated with progress toward SDG 1, SDG 5, SDG 10, SDG 11, and SDG 16. The proportion of synergies in SDG 5, SDG 7, SDG 9, and SDG 10 with other goals was also higher during the study period.

For some SDG interactions, the relations strengthened over 2000–2020. The examination of interactions among 128 different SDGs over the 21-year period showed an increase in the share of synergies for 11 SDG pairs. A significant increase in the proportion of synergies was observed for the 9-2, 9-7, 9–10, 9–13, and 9–14 SDG pairs, suggesting the strengthened association of industry development with clean energy use and environmental conservation in Arctic countries. The 1–7, 2–11, 3–6, 3–7, 13–16, and 14–15 SDG pairs also showed strengthened synergistic associations, with most of the synergy proportions between the goals in each pair increasing by 10–25% from 2016 to 2020, due to the implementation of the 2030 Agenda. These strengthened linkages led to an intensification of relationships in Arctic countries; for example, it was shown that clean energy use promoted poverty reduction, clean water and energy use was more important for the improvement of health, and climate actions required the intervention of more powerful institutions.

The share of trade-offs was relatively lower compared to that of synergies. A relatively higher proportion of trade-offs was observed for the 8–9, 8–11, 3–12, and 10–12 SDG pairs over the 21-year period (Fig. 4). This indicated that Arctic countries need to consider economic growth in terms of the sustainable development of industries and cities, so that responsible production and consumption can counteract the negative effects of growth on health and equality. The share of trade-offs for the 8–9 and 10–12 SDG pairs increased from 6% to 25% and from 12% to 50%, respectively, during the 2015–2020 period. These results imply that more efforts are needed to build a sustainable economic model characterized by both innovation and consumption equality, and that a thorough investigation and relative measures are needed to promote the achievement of SDGs.

**Fig. 5:**
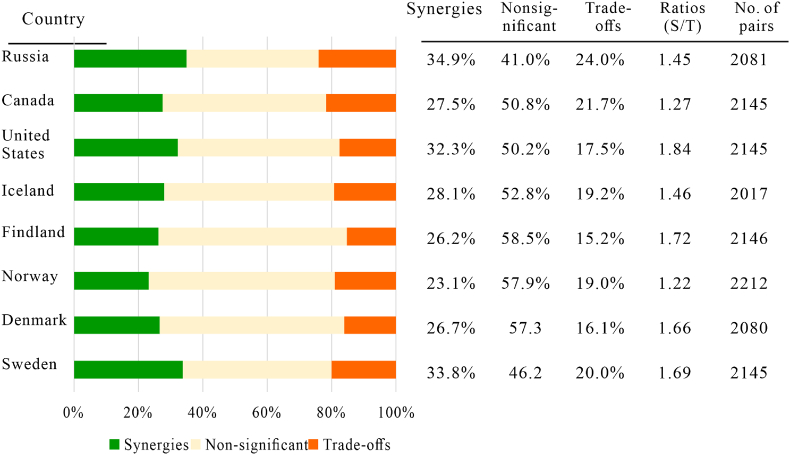
Shares of synergies, non-significant, and trade-offs (including the ratio of synergies to trade-offs) in the total amount of indicator pairs in Arctic countries.

Similarly, at the country level, the positive correlations among SDGs largely outweigh the negative ones for all Arctic countries. As shown in Fig. 5, across countries, the shares of synergies varied between 23.1% for Norway and 34.9% for Russia, while the shares of trade-offs ranged from 15.2% for Finland to 24.0% for Russia. By aggregating the results of all eight countries it was revealed that there were on average approximately 1.5 times more synergies (29.1%) than trade-offs (19.1%). The ratio of synergies to trade-offs also indicated that there were more positive than negative interlinkages across countries (Fig. 5).

Differences in the relationships between goals were also noted. Higher shares of synergies (above 80%) were observed for the indicators of 1–3, 1–4, 1–6, and 3–4 in Russia, indicating the importance of poverty reduction for sustainability in Russia. The United States showed similar values between SDG 11 and other goals, especially 3, 4, 7, and 15, which presents the tightness of health and well-being, education, and clean energy with sustainable cities in the United States. Higher proportions of synergies were observed between SDG 3 and other goals in Canada, indicating a strengthening of the linkage between health improvement and other goals. SDG 9 showed a higher ratio of synergies with other goals in Denmark, and more than 80% of positive correlations were detected between SDG 7 and other goals in Finland and Sweden. These results imply that policy priorities to achieve SDGs need to be differentiated among Arctic countries based on the extent of the SDG synergies and trade-offs observed.

## 4 Discussion

The present study provides deep insights into the progress of Arctic countries toward SDGs during the 2000–2020 period. Part of the results obtained are in line with those reported in previous studies, but a number of differences were also detected. The overall performance of Arctic countries improved during the study period, especially toward SDG 3, SDG 10, and SDG 9. Progress was also observed in SDG 7 and SDG 13, which indicated the commitment of Arctic countries to the mitigation of climate change. The steady leadership of Sweden in comprehensive performance of the 2030 Agenda between 2010 and 2020 was also reflected in the assessment of SDGs established by the UN [3] and EU [8]. The highest scores obtained by Sweden in SDG 3 and SDG 5 were also in line with those reported in previous studies [35], which confirmed the high standard of living of this country and its progress toward a circular and resource-efficient economy. Thus, stronger policy efforts are needed in other Arctic countries to follow the footsteps of Sweden and reach the ambitious set of UN goals by 2030. The relative ranking of the composite performance of Arctic countries was also in line with that reported in previous studies, although some differences were detected. For instance, the GSI score of Denmark was ranked as fourth among Arctic countries in 2016 in our evaluation, while it was ranked as second in Ref. [4]. This discrepancy is mainly due to the fact that, in the present study, the assessment used more high-level indicators and the weight for each indicator was not equalized.

The results of SDG monitoring mainly depend on the indicator used, the normalization function, weighting, and aggregation methods. High-quality data and clear metrics are essential for each country to evaluate where it stands, devise pathways to achieve the goals, and track progress [4]. The contribution of indicators is unequal if their number per goal is uneven. The simple mean aggregation used in previous assessments may be led by strongly related indicators [8]. Thus, these assessments would contribute to obtaining more accurate results, especially considering that resource endowment becomes more important in the context of reverse globalization. There is generally a discrepancy between the expected level of development and a country’s great wealth of natural resources (especially energy). In this study, the index of *Energy imports and total natural resources rent* was added in SDG 7 (*Affordable and clean energy*) and SDG 8 (*Decent work and economic growth*) to intensify and reflect this assumption. The results showed that Norway performed best in terms of progress toward SDG 7, because of its higher energy self-sufficiency and renewable energy proportion. At the same time, Russia was ranked as second in terms of progress toward SDG 8, reporting the highest total natural resources rents, which emphasizes the importance of natural resources in economic development.

The interactions detected between SDGs in this study also provide a useful reference for policy making and a portion of our findings were in accordance with those reported in related studies [33,34]. As concluded in other studies, the interactions between different goals in Arctic countries showed strong positive correlations, indicating that most of the improvements towards these goals were associated with the progress of other goals. A similar conclusion was reached in Ref. [3], where it was found that SDG 1, SDG 3, and SDG 9 had more synergetic relationships with other goals, while SDG 14 had a relatively higher proportion of trade-offs with other goals. However, compared to the interactions observed in a previous global assessment [34], the share of trade-offs was considerably lower in Arctic countries. This is a particularly important finding, because the achievement of the Agenda 2030 crucially depends on whether trade-offs across the entire spectrum of SDGs can be minimized and whether synergies can be maximized over time. The SDG interactions in individual countries were also determined in this study and deep insights were gained.

Although Arctic countries are in a leading position globally in terms of sustainable development (especially the Nordic countries), the hidden “unsustainability” needs to be further considered, such as the high dependency on the rest of the world for energy and virtual water trade. The environmental impact of importing products from producer countries also needs to be taken into consideration and efforts are required to appropriately reduce the intensity of material consumption. In addition, the resilience of progress also should be assessed and strengthened in the future. Lower levels of improvement toward some goals were also noted in 2020—as shown by the significantly reduced scores of SDG 8, SDG 12, and SDG 17 in all Arctic countries—and this was probably due to the COVID-19 pandemic.

The sustainable development of Arctic countries is highly relevant to the whole world. Due to the unique geographical environment of this region, the stability and resilience of its ecosystems is more fragile. The Arctic is currently experiencing an unprecedented level of economic and industrial activity, which may produce short-term benefits but may not yield long-term economic, environmental, or social sustainability for the local communities [29]. The damages associated with unsustainability in the Arctic will exceed by several times those experienced in other regions of the world. Furthermore, the rapidly changing Arctic ecosystems are also interlinked to other parts of the globe [31]. Thus, by pursuing sustainable development, Arctic countries, which are the most affected by climate change, will not only benefit themselves, but also contribute to fulfilling the 2030 Agenda at the global scale [31].

With the deepening impact of climate change and globalization, the sustainable development of Arctic countries will face severe challenges that will also greatly affect the realization of global sustainable development, as changes in the Arctic have repercussions on other regions of the world through natural and socio-economic linkages. Therefore, it is of great significance to clarify the key factors involved in the sustainable development of Arctic countries and the interactions among different indicators, and to construct an assessment framework, so that progress can be monitored and SDGs can be achieved.

## 5 Conclusion

This assessment of performance toward SDGs revealed that Arctic countries vary greatly in terms of their capacity to fulfill the sustainability development agenda and achieve each of the 17 goals. The results also showed that each Arctic country can learn from the others. Sweden showed the best aggregate performance, however, there is still potential for improvement in the fields of natural resources and ecological conservation. Norway made significant progress in decoupling economic growth and human well-being from environmental pressures. Denmark had higher sustainable development levels achieved in poverty reduction, healthy diet, land ecosystem conservation, and good relationships with others, but it needs to make further efforts to improve the health of its ocean ecosystem. The United States should make efforts to reduce inequality and take actions toward energy transition and climate change. Russia showed the worst performance in eight SI indexes but reported the highest score for responsible Consumption and Production.

The relationships of indicators within and among goals in the Arctic countries were also identified in this study. The results showed that synergies dominated over trade-offs among and within SDGs, indicating a strengthening of associations between different goals. However, the relationships varied among different countries. In Russia, poverty reduction presented the largest number of linkages with other goals. Sustainable cities and communities accelerated the SDGs steps of the United States. Progress toward health was tightly associated with the improvement of other goals mostly in Canada. In Denmark, improvements in industry and innovation were accompanied by the progress of numerous other goals. The transformation of the energy system in Finland and Sweden contributed the most to their composite performance toward the achievement of SDGs.

This study provided useful information and evidences for policy makers to define improvement strategies in SDG achieving at national-level in Arctic countries, using much more relatively and precisely indicators and aggregated method. The assessment in this study also contributed a more logical results by applying the neutral weighting scheme and the geometric average aggregation in sustainable progress evaluation. However, there are also some limitations in this study for the availability of data, especially the data on the field of climate change and the response in Arctic countries. In addition, the assessment also need to implement at regional scale in Arctic countries in further studies, for the climate change impact on the sustainable development was much more greatly in these regions.

## Author contribution statement

Qiang Bie: Conceived and designed the experiments; Wrote the paper.

Shijin Wang; Wenli Qiang: Performed the experiments; Analyzed the data.

Xing Ma; Zhengsheng Gu; Nan Tian: Contributed reagents, materials, analysis tools or data; Wrote the paper.

## Funding statement

Shijin Wang was supported by Key Technologies Research and Development Program [2020YFA0608504].

Mr. Qiang Bie was supported by National Natural Science Foundation of China [42101096].

Wenli Qiang was supported by Strategic priority research program of the Chinese Academy of Sciences [XDA19070503].

## Data availability statement

Data will be made available on request.

## Declaration of interest’s statement

The authors declare no competing interests.
